# KPNB1-mediated nuclear translocation of PD-L1 promotes non-small cell lung cancer cell proliferation via the Gas6/MerTK signaling pathway

**DOI:** 10.1038/s41418-020-00651-5

**Published:** 2020-11-02

**Authors:** Wenwen Du, Jianjie Zhu, Yuanyuan Zeng, Ting Liu, Yang Zhang, Tingting Cai, Yulong Fu, Weijie Zhang, Ruochen Zhang, Zeyi Liu, Jian-an Huang

**Affiliations:** 1 Department of Pulmonary and Critical Care Medicine, The First Affiliated Hospital of Soochow University, 215006 Suzhou, China; 2 Suzhou Key Laboratory for Respiratory Diseases, 215006 Suzhou, China; 3 Institute of Respiratory Diseases, Soochow University, 215006 Suzhou, China

**Keywords:** Cancer genetics, Lung cancer

## Abstract

In addition to the role of programmed cell death ligand 1 (PD-L1) in facilitating tumour cells escape from immune surveillance, it is considered as a crucial effector in transducing intrinsic signals to promote tumour development. Our previous study has pointed out that PD-L1 promotes non-small cell lung cancer (NSCLC) cell proliferation, but the mechanism remains elusive. Here we first demonstrated that PD-L1 expression levels were positively correlated with p-MerTK levels in patient samples and NSCLC cell lines. In addition, PD-L1 knockdown led to the reduced phosphorylation level of MerTK in vitro. We next showed that PD-L1 regulated NSCLC cell proliferation via Gas6/MerTK signaling pathway in vitro and in vivo. To investigate the underlying mechanism, we unexpectedly found that PD-L1 translocated into the nucleus of cancer cells which was facilitated through the binding of Karyopherin β1 (KPNB1). Nuclear PD-L1 (nPD-L1), coupled with transcription factor Sp1, regulated the synthesis of Gas6 mRNA and promoted Gas6 secretion to activate MerTK signaling pathway. Taken together, our results shed light on the novel role of nPD-L1 in NSCLC cell proliferation and reveal a new molecular mechanism underlying nPD-L1-mediated Gas6/MerTK signaling activation. All above findings provide the possible combinational implications for PD-L1 targeted immunotherapy in the clinic.

## Introduction

As an important member of the B7 protein family, programmed cell death ligand 1 (PD-L1) is well known for its role in facilitating tumour cells escape from immune surveillance through its binding with receptor programmed cell death-1 (PD-1), which expressed on immune cells [1, 2]. In recent years, despite the considerable clinical improvement that has been achieved with immunotherapy targeting PD-L1/PD-1 checkpoints, a durable response to these therapies was only observed in a minority of patients [3, 4]. In addition to the immunosuppressive properties, accumulated studies have indicated that PD-L1, as a membrane receptor, could transduce intrinsic signals to promote tumour development, differentiation and metabolism [5, 6].

Earlier in 2008, convincing evidence identified that PD-L1 expressed in P815 and Renca cancer cells made them resistant to T cell cytotoxicity even if T cells expressed a signal-null PD-1 [7]. A growing body of evidence has demonstrated that PD-L1 exerts initial oncogenic effects in mediating tumour proliferation, metastasis, autophagy, glucose metabolism and drug resistance [8–10]. It has been reported that in *BRAFV600E* human colon cancer cells, PD-L1 expression was increased, thereby inducing BIM and BIK to enhance chemotherapy-induced apoptosis [11]. In melanoma and ovarian cancer cells, PD-L1 transduced cell-intrinsic signals to regulate immune-independent tumour growth, mTOR signaling and autophagy [12]. Our previous studies have found that PD-L1 can induce NSCLC cells resistant to EGFR-TKIs via TGF-β/Smad signaling pathway [9]. Moreover, we reported that knockdown of PD-L1 inhibited tumour cell proliferation and induced apoptosis in HCC827 and PC9 cell lines [13]. However, how PD-L1 transduced the intrinsic signals and the involved signalosome remained to be illuminated.

The distribution of PD-L1 differs among tumour specimens. PD-L1 is mainly located in the cell membrane and cytoplasm, while it also exists in the nucleus to a less extent. Positive nuclear PD-L1 (nPD-L1) expression is identified in surgically resected renal cell carcinomas, lung cancer and hepatocellular carcinomas tissues [14]. In human oesophageal cancer, nPD-L1 expression is significantly correlated with tumour invasion depth [15]. In colorectal cancer and prostate cancer, nPD-L1 expression in circulating tumour cells has been identified to be significantly correlated with progression-free survival or overall survival, suggesting that nPD-L1 could be a potential prognostic biomarker in cancer patients [16]. In addition, it has been reported that doxorubicin treatment increased nPD-L1 expression via the phosphorylation of AKT in breast cancer [17]. Similarly, PD-L1 translocation played a major role in enhancing tumour cell radioresistance in head and neck squamous cell carcinoma [18]. However, it is still unclear how PD-L1 enters the nucleus and the function of nPD-L1 in NSCLC is rarely explored.

Karyopherin β1, also known as KPNB1, is an important nuclear receptor which involved in shuttling proteins from the cytoplasm to nucleus. It is reported that KPNB1-mediated NUAK1 nuclear translocation was inhibited by oxidative stress [19]. In addition, the presence of KPNB1 is related with tumour progression. In breast cancer, suppression of KPNB1 inhibited cancer cell proliferation by abrogating nuclear transport of Her2 [20]. KPNB1 knockdown can inhibit chronic myeloid leukaemia cell proliferation and induce cell apoptosis via regulation of E2F1 [21]. In colorectal cancer, KPNB1 deficiency suppressed tumour cell growth and metastasis by reducing MET expression [22]. Consistently, our previous study proved that increased KPNB1 expression promoted NSCLC cells proliferation and induced chemoresistance via the PI3K /AKT pathway [23]. These data implicated that KPNB1 could be a potential target for future therapies in cancer; however whether KPNB1 involved in the PD-L1 translocation need to be elucidated.

Tyro3, Axl and MerTK comprise the TAM family of receptor tyrosine kinases (RTKs) [24]. Activation of MerTK signaling by its ligands Growth Arrest-Specific 6 (Gas6) and Protein S1 (PROS1) promoted cell proliferation and metastasis and suppressed the immune response in tumour microenvironment [25–27]. The downstream signaling includes the PI3K/AKT, MAPK/ERK, NF-κB, and JAK/STAT pathways [26, 28]. In glioblastoma, higher expression of MerTK is found to be positively related to tumour TNM stage and malignance, leading to poorer survival for patients [29]. Recent studies demonstrated that MerTK is over-expressed in lymphoma and melanoma and the inhibition of MerTK leads to suppression of tumour growth via AKT and Erk signaling [30, 31]. Moreover, MerTK activation promotes drug resistance to *EGFR* tyrosine kinase inhibitors in NSCLC [32].

In our current study, we have shown that tumour PD-L1 activated intra-tumour cell Gas6/MerTK signals which promoted NSCLC cell proliferation in vitro and in vivo. For the first time, we proved that KPNB1, as an intracellular PD-L1-binding partner, facilitated the nuclear translocation of PD-L1 in NSCLC. Furthermore, nPD-L1, coupled with transcription factor Sp1, transcriptionally regulated Gas6 mRNA synthesis and promoted the secretion of Gas6 to activate MerTK signaling which enhanced NSCLC proliferation. The identification of nPD-L1 redefined its role in cancer development through Gas6/MerTK axis, which provided an alternative view of developing potential therapies for patients who are refractory to PD-L1 targeted immunotherapy.

## Materials and methods

### Patient samples

A total of 24 paired NSCLC tissue specimens were collected in the Department of Pulmonary and Critical Care Medicine, First Affiliated Hospital of Soochow University. All participants were provided with written informed consent at the time of recruitment. All samples were kept at −80 °C for storage. All cases had clinically and pathologically confirmed diagnoses of NSCLC based on the Revised International System for Staging Lung Cancer. The present study was approved by the Ethics Committee of the First Affiliated Hospital of Soochow University. In addition, 52 paired NSCLC tissues and adjacent lung tissues were used for the construction of tissue microarray (Outdo Biotech, Shanghai, China). The detailed information for patients is listed in Tables S1 and S2.

### Haematoxylin-eosin staining (H&E) and immunohistochemical (IHC) assay

The detailed procedures for H&E and IHC analysis were conducted as described in our previous study [33] and also mentioned in our supplementary materials. The antibodies used in our current study were as follows: PD-L1 (2B11D11, ProteinTech Group Inc., Chicago, IL, USA), PD-L1 (28-8, Abcam, Cambridge, UK), PD-L1 (E1L3N, Cell Signaling Technology, Danvers, MA), p-MerTK (ab192649, Abcam, Cambridge, UK) and Ki67 antibody (8D5, Cell Signaling Technology, Danvers, MA).

### Cell culture, transient transfection and establishment of stable cell lines

HCC827 and H1299 cells were purchased from the Cell Bank of the Chinese Academy of Sciences (Shanghai, China). All cell lines were authenticated by STR profiling. Cells were maintained in RPMI-1640 medium, except HEK 293T cells were cultured in DMEM (Sigma, South Logan, UT, USA). All media had 10% foetal bovine serum (Gibco, South America, SA, USA) and 1% antibiotics (Beyotime, Shanghai, China). For siRNA or plasmid transient transfection, cells were seeded in 6-well plates until they reached 40–60% confluence and then transfected with the expression vectors or specific siRNAs targeting Sp1 or MerTK using JetPRIME reagent (Life Technologies). The construction methods of stable PD-L1 knockdown and overexpression cell lines were reported in our previous study [9]. For the generation of KPNB1-overexpressing cells, a 2619-bp fragment of the KPNB1 coding sequence was synthesised (Genewiz, Suzhou, China) and subcloned into the PLVX-IRES-Neo vector using the endonucleases SwaI and NotI. The methods were exactly the same as those performed in the construction of stable PD-L1-overexpressed cells. To generate stable KPNB1 knockout cell lines, gRNAs targeting KPNB1 cloned into plentiCRISPR v2 were synthesised by Genewiz Company. The target sequences were listed in Table S3. Then the KPNB1 gRNA constructs along with the packaging plasmids were co-transfected into HEK 293T cells using Lipofectamine 3000 (Invitrogen) for 48 h. Then we collected the supernatant to infect cancer cells at least three times, followed by puromycin selection. All detailed methods were shown in supplementary materials.

### RNA extraction and quantitative real-time PCR analysis

These procedures were performed as previously mentioned [34]. All PCR primer sequences used for PD-L1, Gas6, MerTK, PROS1, Sp1, CDK1, CDK4 and β-actin mRNA detection; the primers for construction of the Gas6 or Sp1 promoter; and other primers, including Gas6 ChIP primers and primers for KPNB1 stable cell lines or Sp1 overexpression, are listed in Table S3.

### Cellular fractionation, western blot and co-immunoprecipitation assay

Cellular fractions were isolated using the nuclear protein extraction kit (R0050, Solarbio Life Science). Generally, cells were first lysed with cytoplasmic protein extraction buffer, followed by nuclear extraction buffer to obtain the nuclear fraction. In some conditions, after serum starvation for 24 h, PD-L1-overexpressed cells were treated with 3 μM UNC2025 (APExBIO, B8016) or 10 μM LDC1267 (APExBIO, B4893) for 48 h. The PD-L1 knockdown cells were stimulated with 500 ng/ml Gas6 (R&D Systems, 885-GSB) for 24 h. The antibodies used in the analysis were anti-PD-L1 (2B11D11), anti-Tubulin (1E4C11), anti-Lamin B1 (ProteinTech Group Inc., Chicago, IL, USA); anti-Bcl-2(sc-7382), anti-MerTK (sc-365499) (Santa Cruz, CA, USA); anti-KPNB1 (ab2811), anti-p-MerTK (Y749 + Y753 + Y754) (ab14921, Abcam, Cambridge, UK); anti-cyclinD1 (92G2), anti-AKT(11E7), anti-pAKT (Ser473) (D9E), anti-ERK (137F5), anti-pERK (Thr202/Tyr202) (D13.14.4E), anti-Sp1 (D4C3) (Cell Signaling Technology, Danvers, MA, USA); anti-Gas6 (A8545) (Abclonal, Boston, USA); anti-Flag (Sigma, St, Louis, MO, USA); anti-β-actin and anti-mouse or -rabbit secondary antibodies (Cell Signaling Technology). For immunoprecipitation assays, protein lysates were incubated with anti-PD-L1, anti-KPNB1, anti-Flag or the normal IgG antibody at 4 °C overnight with rotation. At day 2, the mixture was further incubated with protein A/G beads or M2 anti-Flag resin at room temperature for 2–3 h. After washing with lysis buffer three times, the beads were boiled and then followed by western blot assay.

### CCK-8 and clonogenic assay

Cells (3 × 10^3^) were seeded in 96-well plates. At 24 h, 48 h and 72 h, Cell Counting Kit-8 solution (Boster, Wuhan, China) was added to each well, and the OD value was determined by measuring the absorbance at 450 nm and 630 nm. For clonogenic assays, cells were cultured for 7–10 days until the formation of foci. Then, the cells were fixed with methanol, stained with Giemsa and counted.

### 5-Ethynyl-2′-deoxyuridine (EdU) assay

The EdU assay kit was purchased from Ribobio Company (C10310-1, Guangzhou, China). Generally, 3 × 10^3^ cells were added to 96-well plates and cultured for 24–48 h to reach 50–60% confluence. The cells were exposed to fresh medium containing 50 µM EdU and cultured in the incubator for an additional 2 h. The cells were fixed with 4% paraformaldehyde and then permeabilized with 0.5% Triton X-100. They were stained with 100 µl Apollo^®^ reaction cocktail for 30 min, followed by 100 µl Hoechst 33342 (5 µg/mL) incubation for 5 min. An Olympus IX-73 inverted microscope (Tokyo, Japan) was used to capture the images.

### Cell cycle and apoptosis analysis

PD-L1-overexpressed cells and the control cells were treated with UNC2025 (3 µM) for 48 h. The cell cycle and apoptosis analysis kit was purchased from Beyotime Biotechnology (Shanghai, China). For the cell cycle analysis, cells were washed with cold PBS and then fixed in 70% ethanol at 4 °C overnight. Then the cells were stained with propidium iodide (PI)/RNase mixture in the dark at 37 °C for 30 min. For the apoptosis assay, both supernatant and cells were harvested, washed, and suspended in binding buffer containing Annexin V/FITC or APC and PI (Beyotime, Shanghai, China). Fluorescence-activated cell sorting (FACS) of stained cells was done in a FACSCalibur system (Beckman Coulter, Brea, CA, USA).

### Immunofluorescence staining

Cells were seeded in 12-well plates that were pre-inserted with glass slides. Twenty-four hours later, when the cells had reached 40–50% confluence, they were washed with PBS. Cells were then fixed with 4% paraformaldehyde for 30 min followed by permeabilization with 0.5% Triton X-100 solution for an additional 20 min. Next, 5% bovine serum albumin was added to function as the blocking buffer. The primary antibodies used in our experiment were anti-PD-L1 (1:200, Abcam) and anti-KPNB1 (1:200, Abcam). The corresponding secondary antibodies tagged with Cy3 and FITC were used (1:500, Beyotime Biotechnology).

### Luciferase reporter assay

H1299-PLVX- and PD-L1-overexpressed cells were transiently transfected with the indicated pGL3-luciferase reporter (500 ng) containing the Sp1 promoter or different truncated regions of the Gas6 promoter, along with the Renilla pRL-TK plasmid (40 ng) set as the inner control. After 24–48 h, the cell lysates were subjected to further luciferase activity examination with a Dual-Luciferase Reporter Assay kit (Promega).

### ELISA

H1299- and HCC827-PD-L1-overexpressed cells were exposed to medium containing 1% FBS for 12 h and then treated with the following inhibitors separately: the autophagy inhibitor 3-methyladenine (1 mM, Selleck, S2767), the protein transport inhibitor brefeldin A (10 ng/ml, APExBIO, B1400), and the exosome secretion inhibitor 5-(N,N-dimethyl)-amiloride DMA (50 nM, APExBIO, C3505). The supernatant was collected from the cultured medium 24 h later. Gas6 level was measured with a human Gas6 ELISA kit (SEA204Hu, USCN Life Sciences, Wuhan, China).

### Mass spectrometry assay

The affinity-purified samples were analysed with the Orbitrap Elite hybrid mass spectrometer (Thermo Fisher) coupled with a Dionex LC according to the instructions. Briefly, the cell lysates from HEK 293T cells transfected with human pcDNA3.1-Flag-PD-L1 plasmid and the control were excised and processed for MS analysis. Samples were loaded onto an enrichment column and separated in an analytical column over 120 min with an LC gradient from 6 to 44% solvent B (80% acetonitrile, 0.1% formic acid). Mass spectra were acquired in the positive-ion mode with an automated data-dependent tandem MS analysis in the CID mode for the 15 most intense ions from each precursor MS scan. Each selected precursor ion was analysed twice in 60 s. The resolution for the precursor ion was set to 120,000, and the MS/MS spectra were collected for the selected precursor ion within a 2 Da mass isolation window.

### Chromatin immunoprecipitation (ChIP) assay

A ChIP-IT kit (Active Motif, Carlsbad, CA, USA) was used to perform the ChIP assay. Briefly, cells were fixed with formaldehyde and then lysed. To precipitate the DNA fragment, 2 μg anti-Sp1 or anti-PD-L1 antibody or normal IgG were used. The DNA-protein complexes were pulled down with magnetic beads and then de-crosslinked. The extracted DNA samples were then amplified with specific Gas6 promoter primers.

### In vivo tumour xenograft animal model

UNC2025 was administered to 4–6-week-old female BALB/c athymic nude mice. The model was established using HCC827-PD-L1 or control cells suspended in PBS and 3 × 10^6^ cells were inoculated subcutaneously into the flanks of 20 nude mice. When the tumour weight reached nearly 100 mm^3^, 10 mice injected with HCC827-PD-L1 cells were randomly divided into two groups with or without UNC2025. And the same was done for the rest 10 mice injected with control cells. UNC2025 was administrated via oral gavage at a dose of 75 mg/kg once a day for 24 days. Tumour weight and tumour volume were monitored every other day. All the animal experiments were carried out in complete compliance with the Guide for the Care and Use of the Experimental Animal Centre of Soochow University.

### Statistical analysis

All experiments were independently performed in triplicate as a minimum. All statistical analyses were done using GraphPad Prism 7.0 (GraphPad, San Diego, CA, USA) and SPSS 17.0 software (SPSS, Chicago, IL, USA). All data were presented as mean ± SD. Significant differences between two groups were assessed by a non-paired Student’s *t* test. Significant differences between three or more groups were analysed using one-way or two-way ANOVA analysis followed by Bonferroni post hoc test. All statistical tests were two-tailed. *P* < 0.05 was set as statistically significant.

## Results

### The role of PD-L1 in mediating NSCLC cell proliferation in vitro

**Fig. 1:**
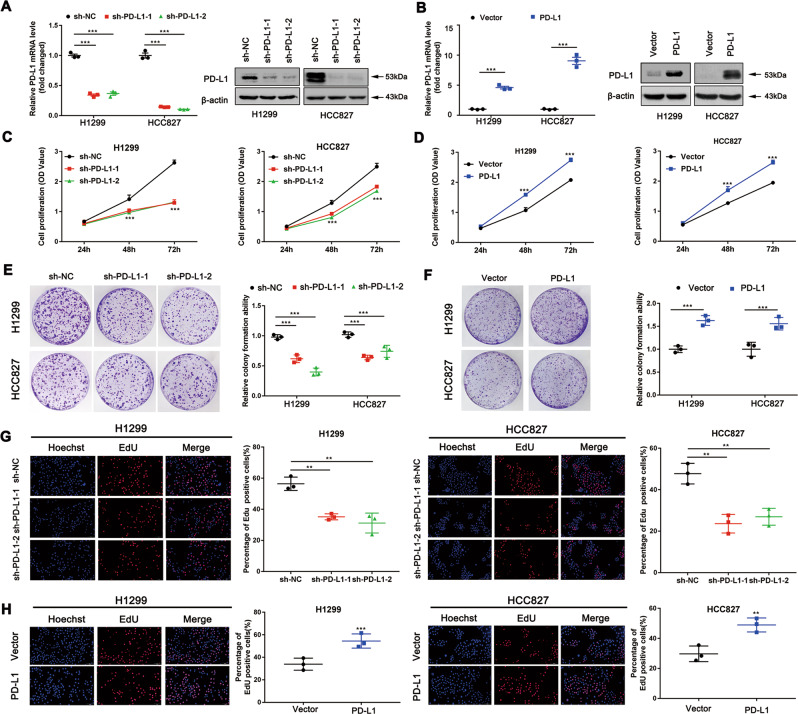
The role of PD-L1 in mediating NSCLC cell proliferation in vitro. **a**, **b** The mRNA and protein levels in H1299 and HCC827 cell lines after PD-L1 knockdown or overexpression. **c**, **d** CCK-8 analysis of H1299 and HCC827 cell viability under PD-L1 silencing or overexpression. **e**, **f** Representative images of the clonogenic assay of H1299 and HCC827 cell proliferation under PD-L1 silencing or overexpression. **g**, **h** Representative images of the EdU assay in H1299 and HCC827 cells with PD-L1 knockdown or overexpression (Scale bar: 2 mm). Data were presented as the mean ± SD. Data were analysed using non-paired Student’s *t* test, one-way or two-way ANOVA analysis followed by Bonferroni’s post hoc test. **P < 0.01, ***P < 0.001 vs. control or as indicated.

To validate the role of PD-L1 in cell proliferation, we first steadily knocked down or overexpressed PD-L1 in H1299 and HCC827 cell lines. The altered PD-L1 mRNA and protein expression levels were confirmed in these two cell lines (Fig. 1a, b). To assess the effect of PD-L1 in cancer cell proliferation, CCK-8 assay was utilised. PD-L1 knockdown inhibited cell growth (Fig. 1c). In contrast, cell growth was significantly enhanced in PD-L1-overexpressed cells (Fig. 1d). We next confirmed this finding via clonogenic assay (Fig. 1e, f). The EdU assay was performed to further verify the above results. Consistently, the percentage of EdU-positive cells was decreased after PD-L1 knockdown (Fig. 1g), while an increased percentage of EdU-positive cells was observed in the PD-L1-overexpressed group (Fig. 1h). Collectively, these data suggested that the presence of PD-L1 promotes NSCLC cell proliferation.

### PD-L1 is positively correlated with p-MerTK expression level in NSCLC tissues

**Fig. 2:**
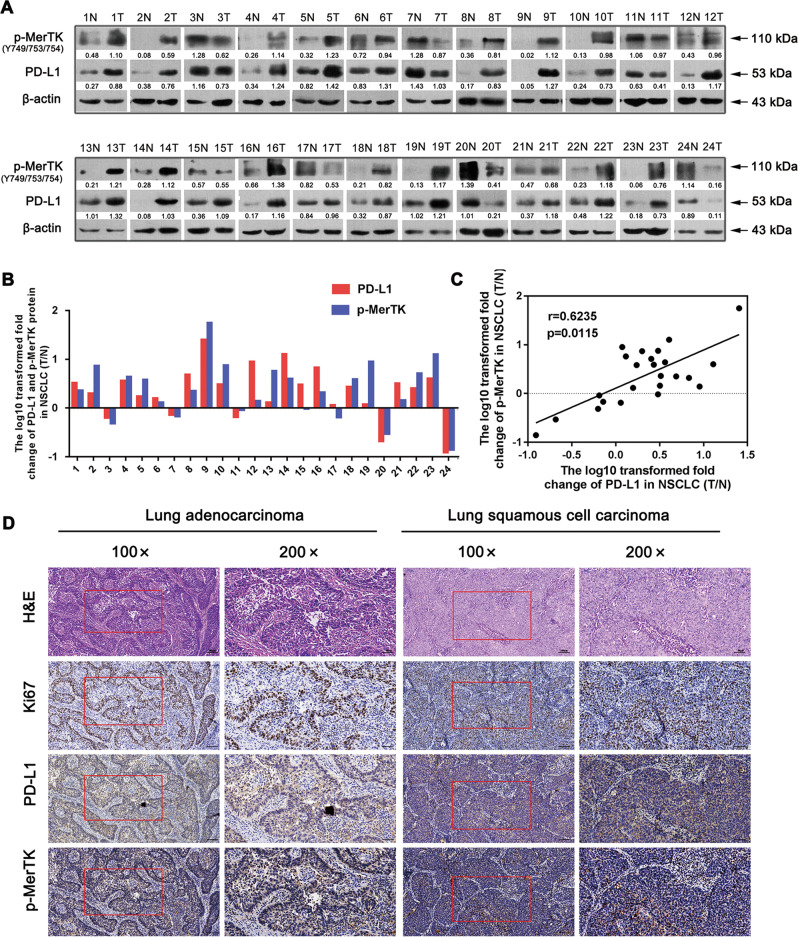
PD-L1 is positively correlated with p-MerTK protein levels in human NSCLC tissues. **a** Western blot assay of PD-L1 and p-MerTK protein levels in 24 paired NSCLC tissues and the corresponding adjacent tissues. **b** Relative quantification of PD-L1 and p-MerTK protein levels in 24 paired NSCLC tissues. The *Y* axis refers to the log10-transformed fold change of the T/N expression ratio. The *X* axis represents sample number. **c** The correlation between PD-L1 and p-MerTK protein levels in 24 paired NSCLC tissues. **d** IHC assay of PD-L1 and p-MerTK protein expression in NSCLC tissues (Scale bar: 100 μm, 50 μm).

To investigate the underlying mechanism, human RTK phosphorylation array was performed to detect the specific tyrosine kinase targets of PD-L1. The data showed that the phosphorylation of MerTK was significantly down-regulated after PD-L1 knockdown (Fig. S1a, c). Other changed kinases were shown in Table S4 and Fig. S1b. We first verified the association between PD-L1 and p-MerTK expression in 24 paired NSCLC tissues and adjacent lung tissues. Among these samples, 17 pairs showed consistently up-regulated PD-L1 and p-MerTK expression in NSCLC tissues. Five pairs demonstrated reduced PD-L1 and p-MerTK expression in NSCLC tissues. Other two cases were presented with non-correlated expression in PD-L1 and p-MerTK (Fig. 2a, b). Further correlation assay showed a positive association between PD-L1 and p-MerTK protein levels (T/N) (Fig. 2c). Moreover, the IHC assay proved that NSCLC tissues with increased PD-L1 expression showed higher p-MerTK expression (Fig. 2d). Individual positive or negative controls were shown in Fig. S2. These findings indicated a positive correlation between PD-L1 and p-MerTK expression levels in NSCLC tissue.

### Tumour intrinsic PD-L1 regulates NSCLC cell proliferation through Gas6/MerTK signaling

**Fig. 3:**
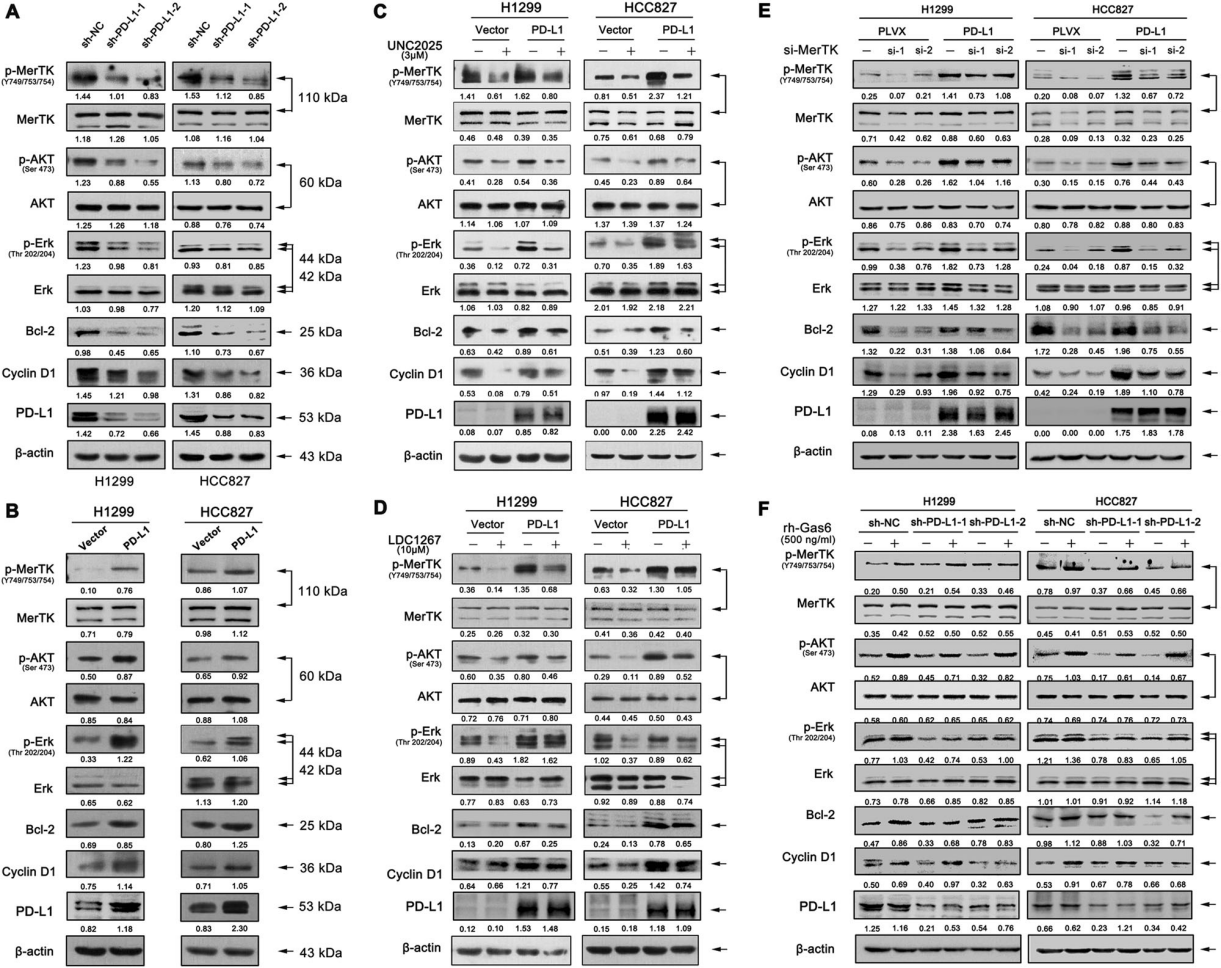
Tumour intrinsic PD-L1 regulates NSCLC cell proliferation through Gas6/MerTK signaling. **a**, **b** Western blot assay of PD-L1, p-MerTK, MerTK, p-AKT, AKT, p-Erk, Erk, Bcl-2 and Cyclin D1 expression in stable PD-L1 knockdown or overexpressed cells when compared to control cells. **c**, **d** PD-L1-overexpressed and control cells were treated with 3 μM UNC2025 or 10 μM LDC1267 for 48 h, then the cell lysates were proceed with western blot assay to test PD-L1, p-MerTK, MerTK, p-AKT, AKT, p-Erk, Erk, Bcl-2 and Cyclin D1 protein levels. **e** Western blot assay of the above proteins in PD-L1 overexpressing cells after knocked down with two separate si-MerTKs. **f** Western blot assay of p-MerTK, MerTK, p-AKT, AKT, p-Erk, Erk, Bcl-2 and Cyclin D1 protein levels in PD-L1 stable knockdown cells after stimulated with 500 ng/ml rh-Gas6 for 24 h.

We also verified the association between PD-L1 and p-MerTK expression in NSCLC cell lines. The expression of p-MerTK was down-regulated in PD-L1 knockdown cell lines. Meanwhile, p-AKT, p-Erk, Cyclin D1, and Bcl-2 were markedly reduced, while the total AKT and Erk levels were not changed (Fig. 3a). In contrary, PD-L1 overexpression up-regulated p-MerTK, p-AKT, p-Erk, Cyclin D1 and Bcl-2 expression levels in both H1299 and HCC827 cell lines (Fig. 3b). To further validate the role of MerTK and its associated downstream signaling pathway in PD-L1-mediated cell proliferation, PD-L1-overexpressed and control cells were treated with the MerTK selective inhibitor UNC2025. PD-L1-overexpression rescued UNC2025-induced decreased percentage of cells in the G0/G1 and S phases and increased percentage of cells in the G2/M phase when compared to control group (Fig. S3a). Further, we found that UNC2025 treatment resulted in an increase in cell apoptosis, while PD-L1 overexpression reduced the UNC2025-mediated apoptosis as evidenced by a lower cell apoptosis rate (Fig. S3b). In addition, PD-L1 overexpression rescued the decreased expression levels of p-MerTK, p-AKT, p-Erk, Cyclin D1 and Bcl-2 after UNC2025 treatment, which was in accordance with the flow cytometry results (Fig. 3c). Moreover, we observed similar results in cells which were treated with two individual MerTK specific siRNAs (Fig. 3e). LDC1267 is a widely used inhibitor for TAM signaling pathway. We found that PD-L1 overexpression rescued the reduced expression levels of p-MerTK, p-AKT, p-Erk, Cyclin D1 and Bcl-2 after LDC1267 treatment when compared to control cells (Fig. 3d). To further confirm these findings, we activated MerTK with rh-Gas6 in PD-L1 knockdown cell lines. PD-L1 knockdown blocked Gas6-induced MerTK signaling activation compared to control cells (Fig. 3f). These results strongly suggested that PD-L1 controls tumour cell proliferation at least partially by regulating the cell cycle and apoptosis via MerTK signaling pathway.

### Inhibition of MerTK signaling blocks PD-L1-overexpression induced tumour growth in murine xenograft model

**Fig. 4:**
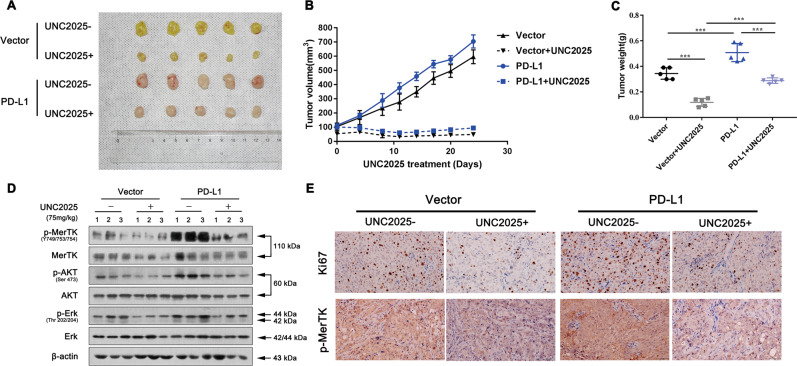
Inhibition of MerTK signaling blocks PD-L1-overexpression induced tumour growth in murine model. **a** Representative images of tumours in these four groups. See the Methods for details. **b**, **c** Tumour volume and weight in the PD-L1overexpression and control groups with or without UNC2025 treatment. **d** Western blot assay of p-MerTK, MerTK, p-AKT, AKT, p-Erk, Erk, Bcl-2 and Cyclin D1 protein levels in these four groups. **e** IHC assay of KI-67 and p-MerTK expression (Scale bar: 100 μm). Data were presented as the mean ± SD. Data were analysed using two-way ANOVA analysis followed by Bonferroni’s post hoc test. ***P < 0.001 vs. control or as indicated.

Then we extended our study to murine subcutaneous xenograft models by administering UNC2025 in vivo. HCC827 cells with stable PD-L1 overexpression or control cells were inoculated into BALB/C athymic mice. Tumour growth was significantly inhibited in mice treated with 75 mg/kg UNC2025 in both PD-L1-overexpressed mice and control mice. However, the tumour sizes were much larger in the PD-L1-overexpressed mice compared to control mice when both groups were administrated with equal amount of UNC2025 (Fig. 4a, b). The changes in tumour weight were in line with the tumour volume (Fig. 4c). The tissues resected from the xenograft tumour were then lysed for western blot assay. PD-L1 overexpression rescued the decreased activation of MerTK and downstream AKT and Erk signaling after UNC2025 treatment (Fig. 4d). We then confirmed the above findings through analysing the p-MerTK and Ki67 expression levels in these groups (Fig. 4e). Collectively, all above data indicated that tumour intrinsic PD-L1 promotes cell proliferation via Gas6/MerTK signaling in vitro and in vivo.

### Tumour intrinsic PD-L1 regulates Gas6 transcription and secretion

**Fig. 5:**
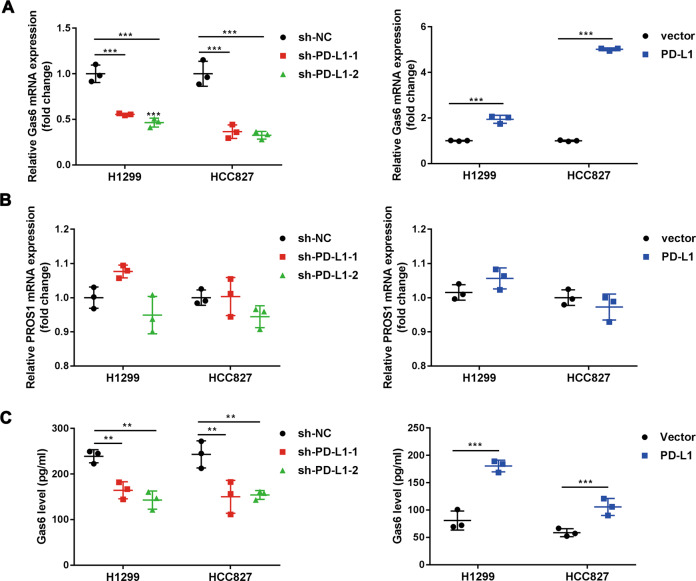
Tumour intrinsic PD-L1 promotes Gas6 transcription and secretion. **a**, **b** qRT-PCR analysis of Gas6 and PROS1 mRNA expression levels in stable PD-L1 knockdown and overexpression cell lines. **c** The basal secretion level of Gas6 in PD-L1 stable cell lines and control cells. Data were presented as the mean ± SD. Data were analysed using non-paired Student’s *t* test, one-way ANOVA analysis followed by Bonferroni’s post hoc test. **P < 0.01; ***P < 0.001 vs. control or as indicated.

Next, we sought to determine the mechanism that how MerTK phosphorylation was regulated by PD-L1. It is well known that MerTK signaling is activated via binding with its ligands Gas6 and PROS1. Therefore, we tested whether PD-L1 could regulate Gas6 or PROS1 expression. QRT-PCR data showed that Gas6 mRNA expression was down-regulated in PD-L1 knockdown cell lines, while PD-L1 overexpression up-regulated Gas6 expression at the transcriptional level (Fig. 5a). In term of PROS1, no significant changes were found after PD-L1 knockdown or overexpression (Fig. 5b). Interestingly, ELISA data found that manipulation of PD-L1 expression regulated Gas6 secretion (Fig. 5c). It has been reported that Gas6 can bind to phosphatidylserine-positive vesicles, such as exosomes, and act as exosome-derived ligands to activate TAMs [35]. We verified this mechanism by applying the autophagy inhibitor 3-MA, the Golgi apparatus transport inhibitor brefeldin A and the exosome biogenesis inhibitor DMA. Only DMA treatment showed a blocking effect on the secretion of Gas6, suggesting that Gas6 can be secreted via the exosome (Fig. S4). All above data suggested that PD-L1 promoted Gas6 transcription and secretion, which may contribute to MerTK activation.

### KPNB1 is critical for PD-L1 nuclear translocation

**Fig. 6:**
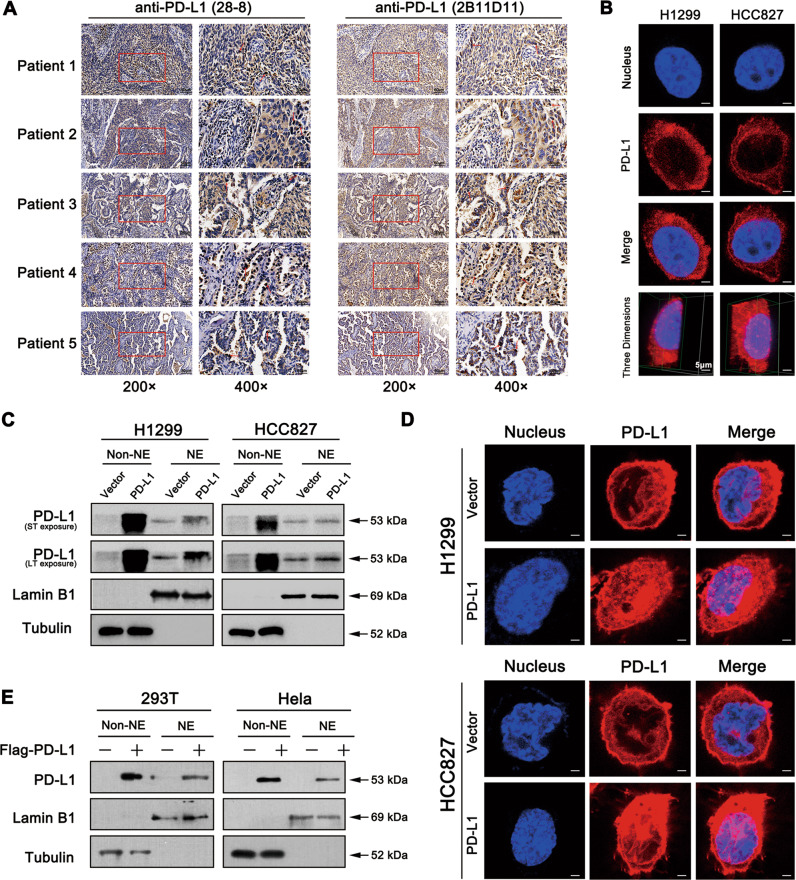
PD-L1 overexpression can promote its nuclear translocation. **a** IHC assay of nPD-L1 staining with two separate anti-PD-L1 clones (28-8; 2B11D11) on the serial histological section of 24 paired NSCLC tissues. Five cases were selected as representative images. And the red arrows indicated positive nPD-L1 staining (Scale bar: 50 μm). **b** Immunofluorescence staining of nPD-L1 location in H1299 and HCC827 cell lines analysed by confocal microscopy (Scale bar: 5 μm). **c** PD-L1-overexpressed cells and control cells were subjected to cellular fractionation followed by western blotting for PD-L1. Tubulin and Lamin B1 were the endogenous markers for cytosolic and nuclear proteins. **d** Immunofluorescence staining of nPD-L1 location in PD-L1-overexpressed cells compared to control cells (Scale bar: 5 μm). **e** HEK293T and Hela cells were transfected with the Flag-PD-L1 or control plasmids and then subjected to western blot. NE nuclear extract.

PD-L1 is widely recognised to be located in the cytoplasm and cell membrane. A few studies have also shown that the nPD-L1 was observed in surgically resected lung adenocarcinomas and lung squamous cell carcinomas tissues [14]. Based on our finding that manipulation of PD-L1 regulated the expression and secretion of Gas6, and this raised the possibility that PD-L1 might enter the nucleus to promote Gas6 transcription. First, we used three different clones of anti-PD-L1 antibodies to detect nPD-L1 expression. In addition to 2B11D11 clone used in current study, another universally recommended anti-PD-L1 antibody (clone 28-8) was utilised to stain the same tissue sections (Fig. 6a). It was reported that nPD-L1 could be detected by using anti-PD-L1 antibody (clone E1L3N) [14]. So next we sought to detect nPD-L1 in our 52 paired NSCLC tissue microarray using clone E1L3N antibody. Among these 52 paired tissues, 17 cases showed positive nPD-L1 staining. And we randomly selected representative 6 cases to show nPD-L1 expression (Fig. S5a). Taken together, we found positive nPD-L1 expression in NSCLC tissues using three different anti-PD-L1 antibodies, which is in line with the published results [14]. Confocal microscopy analysis showed nPD-L1 location in NSCLC parental cells (Fig. 6b). We further verified the presence of nPD-L1 by separating the cytoplasmic (non-NE) and nuclear (NE) fractions from PD-L1-overexpressed and control cells. We also noticed that nPD-L1 expression level was increased in PD-L1-overexpressed cells (Fig. 6c), which was further supported by confocal microscopy data (Fig. 6d). Similar results were obtained from HEK293T and Hela cells, which were transfected with Flag-PD-L1 plasmid (Fig. 6e).

Recently, it was reported that external factors, such as hypoxia, irradiation, and chemotherapeutic agents had effects on the distribution of PD-L1 within the cells [16, 17]. To test whether PD-L1 can enter the nucleus of NSCLC cells under the treatment of paclitaxel and cisplatin, cytoplasmic and nuclear fraction of treated cells was isolated and then subjected to western blot analysis. Interestingly, paclitaxel significantly promoted the nuclear translocation of PD-L1 compared to that of cisplatin (Fig. S5b, c).

**Fig. 7:**
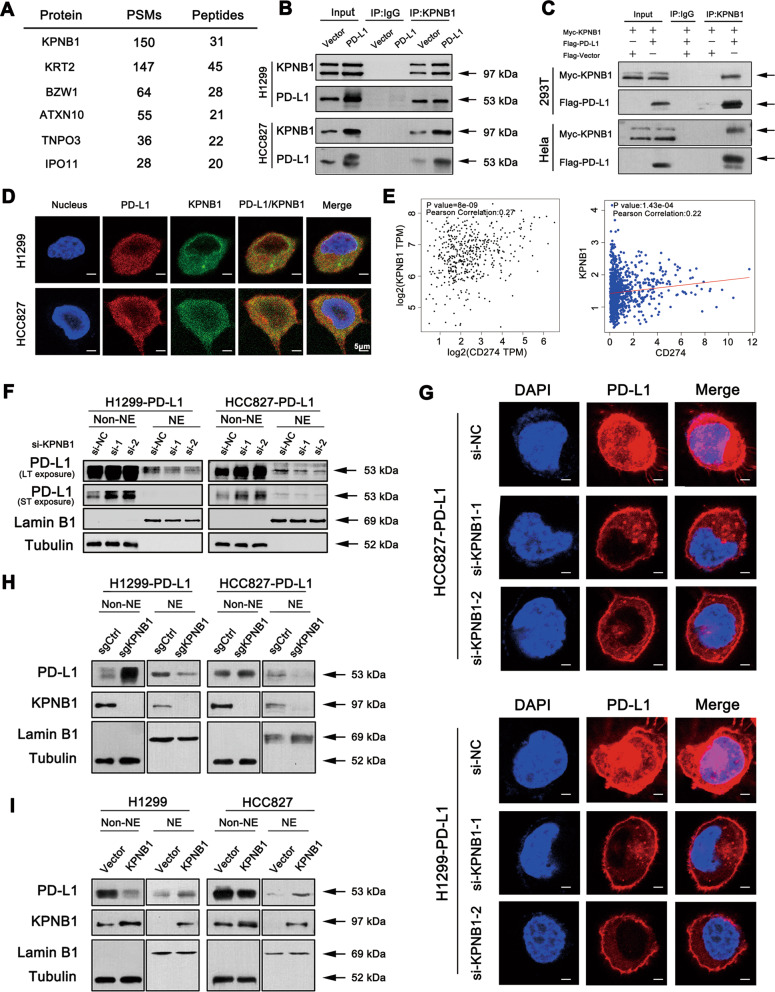
KPNB1 is responsible for PD-L1 nuclear translocation. **a** The mass spectrometry assay of the potential PD-L1-interacting proteins. **b** Endogenous KPNB1 and PD-L1 interaction were detected in NSCLC cells using anti-KPNB1 and anti-PD-L1 antibodies for co-IP and Western blot assays. **c** HEK293T and Hela cells were transfected with the indicated plasmids. Western blot and co-IP assays were performed to assess the interaction between PD-L1 and KPNB1. **d** The cellular location of PD-L1 and KPNB1 examined by confocal microscopy (Scale bar: 5 μm). **e** Correlation assay of PD-L1 and KPNB1 expression in NSCLC patient samples based on linkedomics and GEPIA database. **f**, **h** Western blot assay of cytoplasmic and nuclear PD-L1 expression after KPNB1 knockdown with two specific siRNAs or knockout with CRISPR-Cas9 gene editing system. **g** Immunofluorescence staining of nPD-L1 location after KPNB1 knockdown in PD-L1-overexpressed cells (Scale bar: 5 μm). **i** Western blot assay of nPD-L1 expression in KPNB1-overexpressed cells.

To gain insight into the underlying mechanism of PD-L1 nuclear import process, we performed mass spectrometry analysis in human HEK 293T cells to determine the potential interacting partners of PD-L1. We found that KPNB1 was one of the most strongly associated proteins with PD-L1 (Figs. 7a). To further test this finding, co-immunoprecipitation assay was performed and endogenous interaction of PD-L1-KPNB1 complex was confirmed in H1299 and HCC827 cells (Fig. 7b and S6a). We further co-expressed myc-tagged KPNB1 and Flag-tagged PD-L1 in HEK 293T cells to verify the above finding. PD-L1 was detected in KPNB1 immunoprecipitates, indicating that PD-L1 interacted with KPNB1 (Fig. 7c). As shown in Fig. 7d, KPNB1 was co-localised with membrane, cytoplasmic and nuclear PD-L1 via confocal microscopy analysis (Fig. 7d). Public database showed also that KPNB1 expression was positively correlated with PD-L1 in NSCLC tissues (Fig. 7e). To investigate whether the process of PD-L1 nuclear translocation was affected by KPNB1, two siRNAs were individually used to knockdown the expression of KPNB1, and PD-L1 nuclear translocation was inhibited (Figs. 7f and S6b), which was further supported by immunofluorescence assays (Fig. 7g). In addition, similar result was obtained in PD-L1-overexpressed cell lines in which KPNB1 was genetically modified using CRISPR/Cas9 (Fig. 7h). In contrast, overexpression of KPNB1 promoted PD-L1 nuclear accumulation (Fig. 7i). Taken together, the above data suggested that KPNB1 is responsible for PD-L1 nuclear transport.

### NPD-L1 mediates Gas6 transcription via interaction with the transcription factor Sp1

**Fig. 8:**
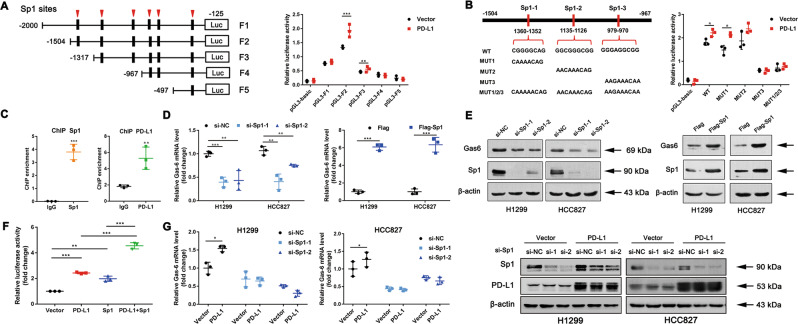
PD-L1 cooperates with SP1 to regulate Gas6 transcription. **a**, **b** The results of the luciferase reporter assay in H1299-PD-L1-overexpressed cells and control cells after transfection with a set of luciferase reporter plasmids containing different regions of the Gas6 promoter or mutants at three SP1 binding sites for 24 h. **c** ChIP assays were performed to detect PD-L1 and Sp1 binding to the Gas6 promoter in H1299-PD-L1-overexpressed cells and control cells. **d**, **e** The mRNA and protein levels of Gas6 in H1299 and HCC827 cells after transient transfection with Sp1 siRNAs or Sp1-overexpressing plasmids. **f** The luciferase activity of the Gas6 promoter in 293T cells transfected with control vector, PD-L1 vector, Sp1 vector or both PD-L1 and Sp1 vectors. **g** H1299- and HCC827-PD-L1-overexpressed cells were silenced with si-Sp1 or si-NC (control). Data were presented as the mean ± SD. Data were analysed using non-paired Student’s *t* test, one-way or two-way ANOVA analysis followed by Bonferroni’s post hoc test. *P < 0.05; **P < 0.01; ***P < 0.001 vs. control or as indicated.

Although nPD-L1 was reported to be discovered in the nucleus, no studies have been conducted to explore the function of nPD-L1 as a transcription factor. So next we speculated whether nPD-L1 can cooperate with various transcription factors to promote Gas6 transcription. First, we segmented the −2000 kb 5′-region of the Gas6 promoter and constructed the corresponding luciferase reporter plasmids. Sequence range from −1504 to −967 bp in the Gas6 promoter region showed increased luciferase activity in PD-L1-overexpressed cell line, suggesting this region is indispensable for the PD-L1-induced Gas6 promoter activity (Fig. 8a). Sequence analysis indicated that three potential Sp1 binding sites were located at the region from −1504 to −967. Mutation of the third or all Sp1 binding sites abrogated the PD-L1-induced luciferase activity, suggesting Sp1 is essential for PD-L1-induced Gas6 promoter activity (Fig. 8b). Moreover, ChIP assay showed significant enrichment of both PD-L1 and Sp1 at the Gas6 promoter (Fig. 8c). We next tested whether Sp1 regulated Gas6 expression. Sp1 knockdown led to the reduced Gas6 expression, whereas overexpression of Sp1 increased Gas6 mRNA and protein expression levels (Fig. 8d, e). Furthermore, we demonstrated that the PD-L1-induced up-regulation of Gas6 mRNA expression was abrogated in the Sp1 knockdown condition but not with the control siRNA (Fig. 8g). Consistent with the above result, combination of PD-L1 and Sp1 synergistically enhanced the Gas6 promoter activity than applying PD-L1 or Sp1 alone (Fig. 8f). Together, all the above data showed that PD-L1 regulates the expression of Gas6 by cooperating with transcription factor Sp1.

### Gas6/MerTK signaling promotes NSCLC cell proliferation

**Fig. 9:**
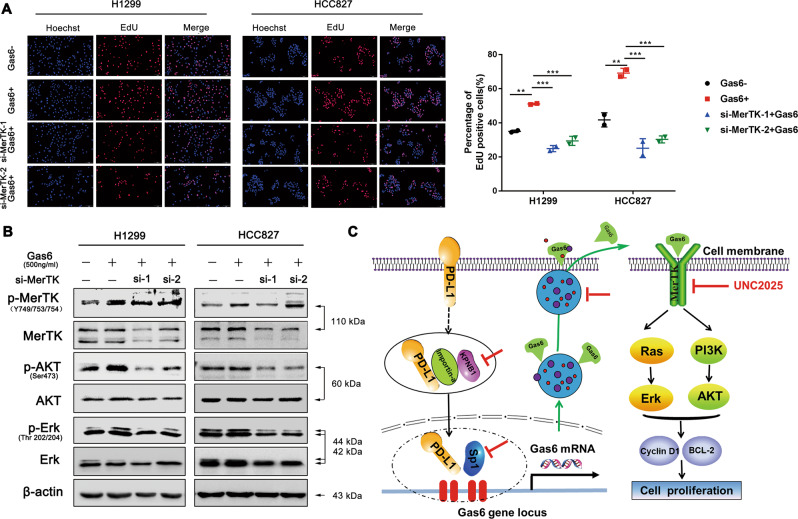
Gas6/MerTK signaling promotes NSCLC cell proliferation. **a** EdU assay was performed in H1299 and HCC827 cells transfected with si-MerTK or si-NC with or without Gas6 stimulation for 24 h (Scale bar: 2 mm). **b** Western blot assay of p-MerTK, MerTK, p-AKT, AKT, p-Erk and Erk. **c** The proposed mechanistic model underlying the nPD-L1-mediated Gas6/MerTK signaling stimulating NSCLC cell proliferation. Data were presented as the mean ± SD. Data were analysed using one-way ANOVA analysis followed by Bonferroni’s post hoc test. **P < 0.01; ***P < 0.001 vs. control or as indicated.

We have previously reported that PD-L1 can regulate Gas6 transcription and secretion, so next we mainly focussed on the role of Gas6/MerTK signaling it played in cell proliferation. Treatment of exogenous rh-Gas6 promoted the cell proliferation in H1299 and HCC827 cell lines, and the effect of cell proliferation was mediated by the engagement of MerTK signaling as knockdown of MerTK inhibited Gas6-induced proliferation (Fig. 9a). Western blot results revealed increased protein levels of p-MerTK, p-AKT and p-Erk after Gas6 stimulation, while this up-regulation was inhibited after silencing MerTK expression (Fig. 9b).

## Discussion

The role of PD-L1 in mediating tumour cells escape from immune surveillance has been widely discussed. Accumulating clinical trials have demonstrated the promising outcome in prolonging the long-term and durable overall survival in advanced NSCLC patients [36–38]. However, only 20–30% of NSCLC patients responded to the immunotherapy targeting PD-1/PD-L1 checkpoints [39]. And some patients even with higher PD-L1 expression still cannot benefit from the immunotherapy. Recent studies have also highlighted the role of tumour intrinsic PD-L1 in promoting cell proliferation, metastasis, drug resistance, metabolism and other effects independent of the immune system [8–13]. Here we first demonstrated that partial PD-L1 translocated into the nucleus to further regulate Gas6 expression and thus activate MerTK signaling pathway to promote NSCLC cell proliferation.

NPD-L1 has been implicated in mediating the drug resistance of breast cancer cell and functions as a prognostic indicator in prostate cancer and colorectal cancer patients [16, 17]. It is also reported that nPD-L1 competes with WAPL for binding to PDS5B, and regulates breast cancer cell sister chromatid cohesion [40]. Here, our study confirmed the positive nPD-L1 expression in NSCLC tissues and cell lines. Recently, the underlying mechanism behind PD-L1 nuclear translocation has been explored in breast cancer. Under hypoxia treatment, p-Stat3 physically interacts with PD-L1 and facilitates its nuclear translocation [41]. Another report found that deacetylation of PD-L1 at Lys 263 by HDAC2 triggers nuclear translocation by interacting with multiple proteins that are involved in endocytosis and nuclear import like HIP1R, vimentin [42]. Here in our study, we found that KPNB1 was the most relevant protein with PD-L1. Moreover, KPNB1 participates in regulating the cell cycle, which is essential for cancer cell proliferation [43–45]. The Oncomine database (https://www.oncomine.org/) indicated higher KPNB1 expression in lung cancer tissues, implying poorer overall survival and progression-free survival (http://www.kmplot.com/) (Fig. S7). We found that knockdown of KPNB1 blocked PD-L1 nuclear import, implying that KPNB1 was critical for PD-L1 nuclear translocation. However, further studies are needed to better clarify the functional-binding domains between PD-L1 and KPNB1. Besides, we need to determine the source of nPD-L1 whether from cell membrane or from cytoplasm.

No studies have shown that PD-L1 contains a DNA-binding domain and thus functions as a transcription factor. Recent new findings showed that nPD-L1 and p-Y705-Stat3 acted as co-activators in regulating GSDMC mRNA expression and then switched apoptosis to pyroptosis [41]. Moreover, nPD-L1 interacted with STAT3, c-Jun to govern immune-response-related genes expression and thereby modulated the anti-tumour immune response [42]. Sp1 is well identified as a common transcription factor that recognises the GC-rich DNA sequences in the promoter regions of various genes [46, 47]. Here our data demonstrated strong Sp1 enrichment at the promoter region of Gas6. And Sp1, together with nPD-L1, acted as co-activators to regulate Gas6 transcription. While there is a possibility that other transcription factors may participate in Gas6 transcription since Sp1 is reported to have overlapping function with other Sp/KLF family members, such as Sp3 [48]. To our surprise, a connection between PD-L1 and Sp1 was found as PD-L1 can regulate Sp1 at mRNA and protein levels (Fig. S8). It is reported that the interaction between proteins and Sp1 depends on the Sp1 zinc finger DNA-binding domain (ZFDBD) [49]. Therefore, whether PD-L1 directly interacts with the ZFDBD of Sp1 still remains unclarified. Surprisingly, we further found that the mRNA levels of Sp1-regulated targets *CDK1*, *CDK4* and *HOGG1* were up-regulated after PD-L1 overexpression, suggesting that PD-L1 might regulate other Sp1 targets as well [50, 51]. Therefore, we speculate that PD-L1 could interact with Sp1 to form protein complex to function as transcriptional regulators.

MerTK was first recognised for its role in mediating the phagocytosis of apoptotic cells, which negatively regulating the immune system [52]. Recent studies also indicated high MerTK expression in various tumour tissues, and the activation of MerTK signaling contributed to tumour progression [53]. Studies showed that PS-induced MerTK activation led to PD-L1 up-regulation in breast cancer cells [54]. Another study suggested that MerTK overexpression in epithelial cancer cells drives efferocytosis via increasing PD-L1 expression [55]. Although these studies showed that MerTK induced PD-L1 up-regulation, interestingly, our data found that MerTK activation was regulated by PD-L1 in both NSCLC cell lines and patient samples. Moreover, we identified that PD-L1 can regulate cell cycle and apoptosis to promote cell proliferation via the MerTK signaling pathway. In addition, it would be interesting to clarify whether nPD-L1-mediated Gas6/MerTK signaling activation may account for the compromised immunotherapy response which targeting PD-L1/PD-1 checkpoints in patients with higher PD-L1 levels, which needs to be further investigated.

In summary, our study for the first time demonstrated that PD-L1 can regulate NSCLC proliferation via Gas6/MerTK signaling. Furthermore, we identified KPNB1 as the cytoplasm-to-nucleus shuttle for PD-L1 and established a novel role for nPD-L1 in promoting Gas6 transcription via the transcription factor Sp1 (Fig. 9c). These data redefine the function of nPD-L1 in tumour, and we speculate that targeting KPNB1 or MerTK signaling pathway in combination with PD-L1 immunotherapy may improve the clinical outcome of NSCLC patients.

## Supplementary information

Figure S1

Figure S2

Figure S3

Figure S4

Figure S5

Figure S6

Figure S7

Figure S8

Table S1

Table S2

Table S3

Table S4

Supplementary figure and table legends

Suplementary materials and methods
