# Supplementary figures and images for: KPNB1-mediated nuclear translocation of PD-L1 promotes non-small cell lung cancer cell proliferation via the Gas6/MerTK signaling pathway
Source: Cell Death Differ. 2020 Nov 2;28(4):1284–300. doi: 10.1038/s41418-020-00651-5 (PMC8027631; doi:10.1038/s41418-020-00651-5)

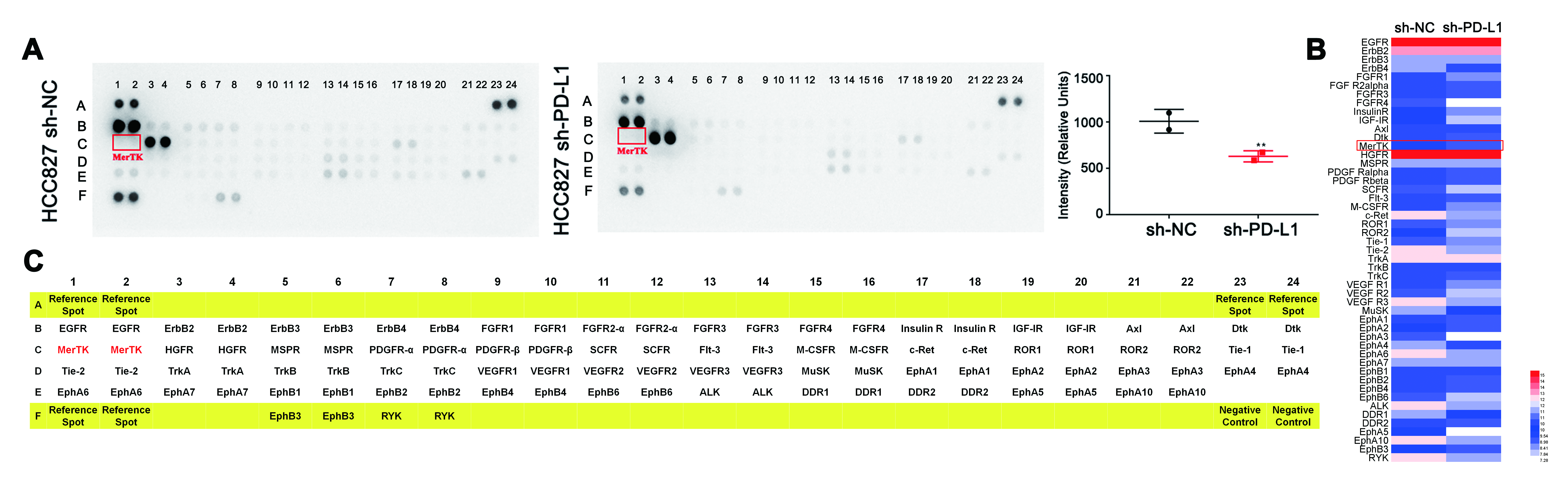

Supplement: Supplementary file 1 — Figure S1 [file 41418_2020_651_MOESM1_ESM.tif]

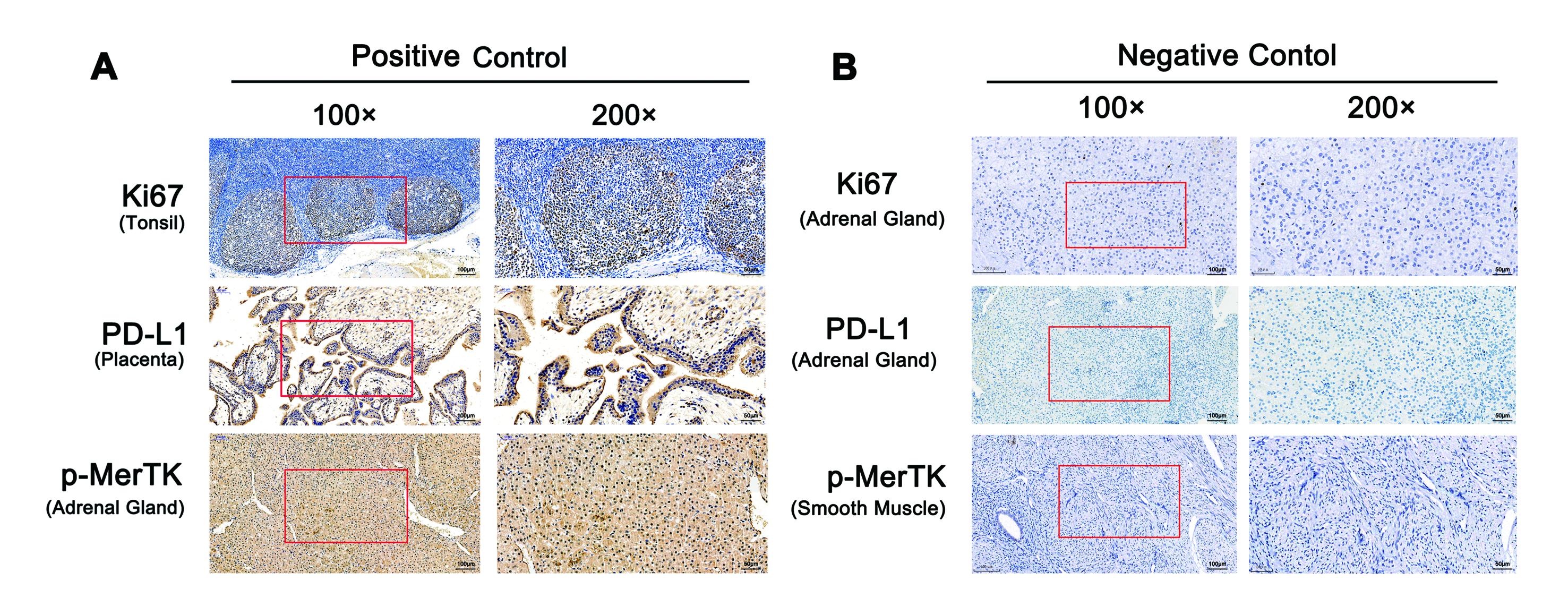

Supplement: Supplementary file 2 — Figure S2 [file 41418_2020_651_MOESM2_ESM.tif]

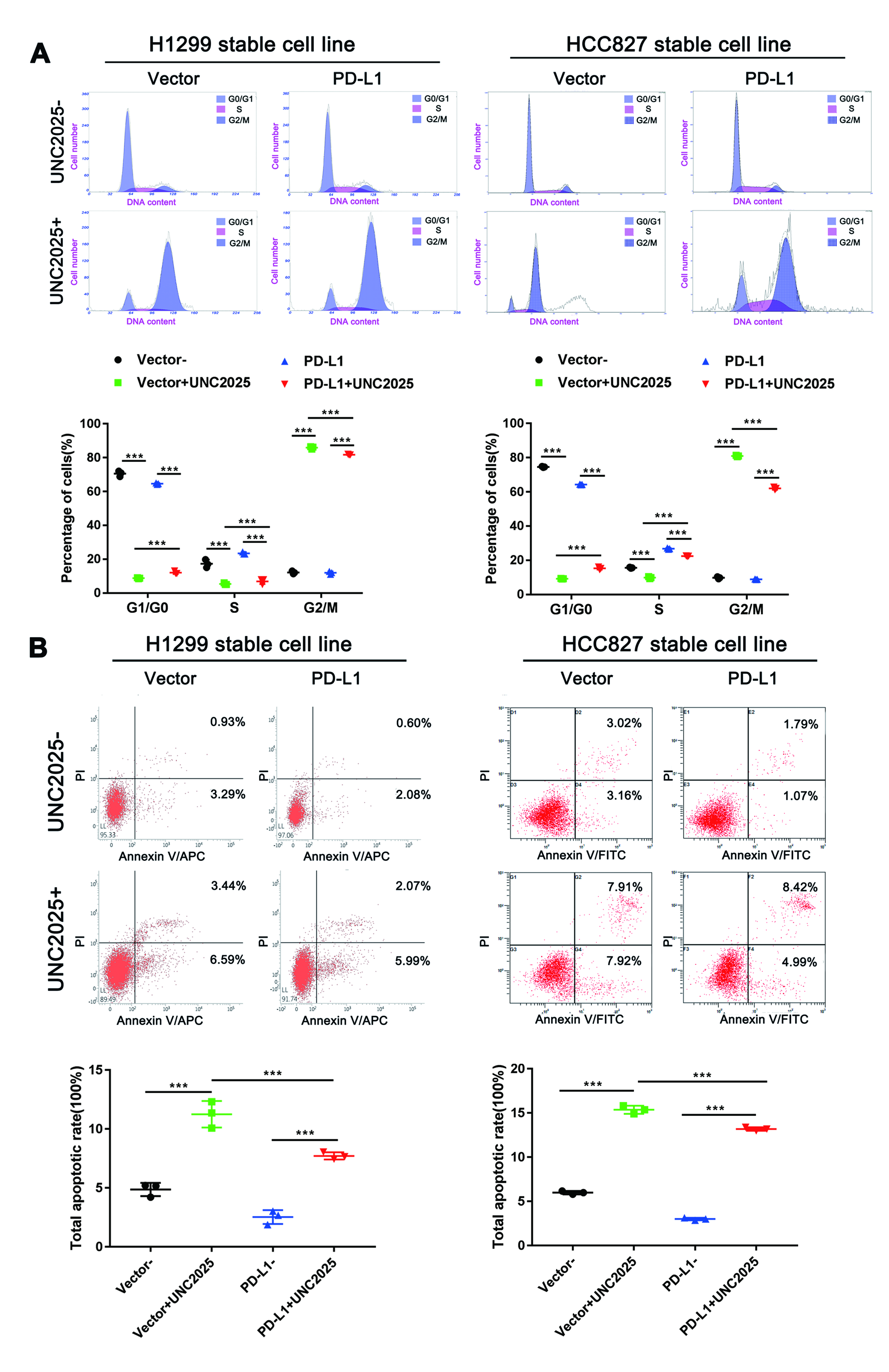

Supplement: Supplementary file 3 — Figure S3 [file 41418_2020_651_MOESM3_ESM.tif]

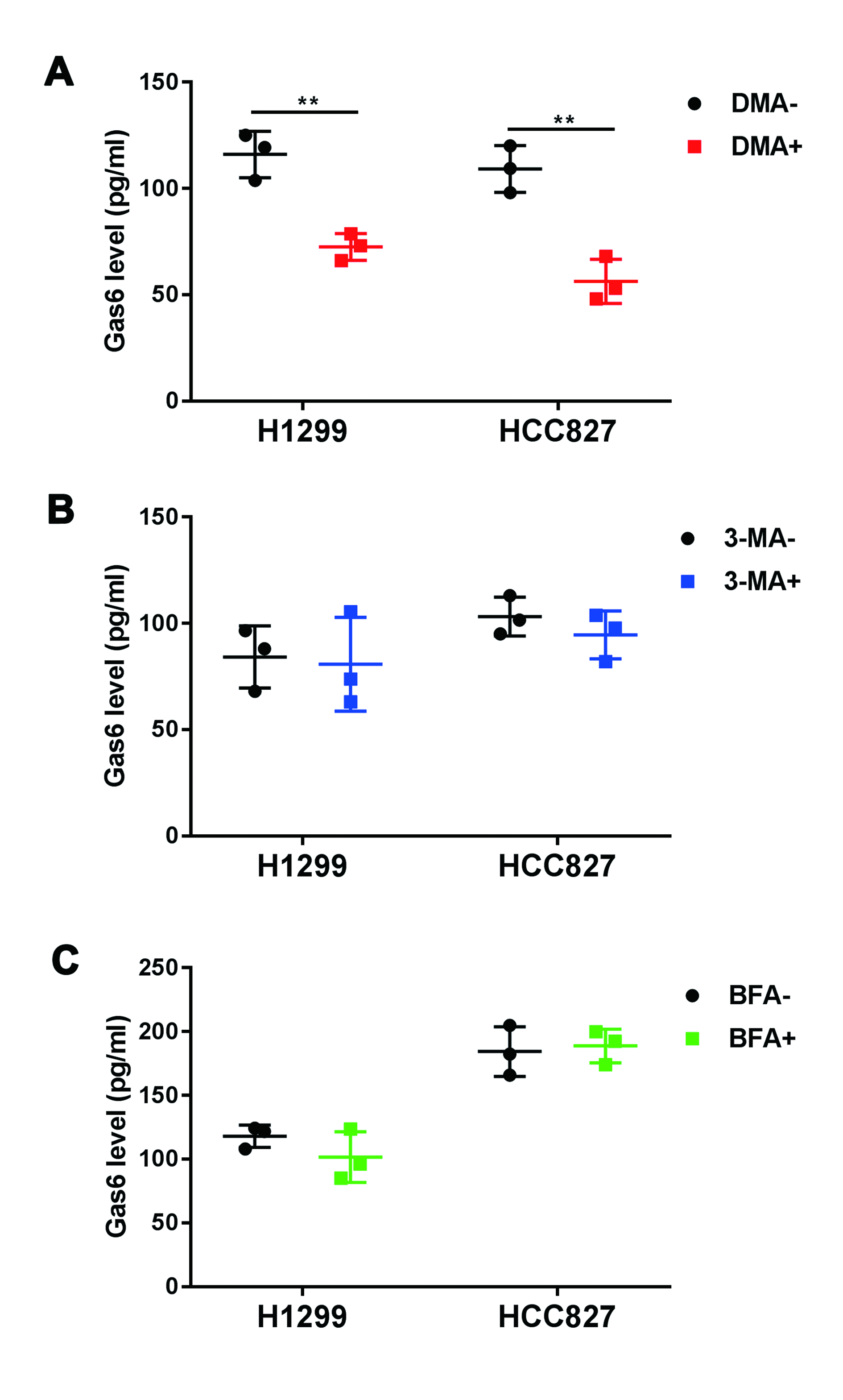

Supplement: Supplementary file 4 — Figure S4 [file 41418_2020_651_MOESM4_ESM.tif]

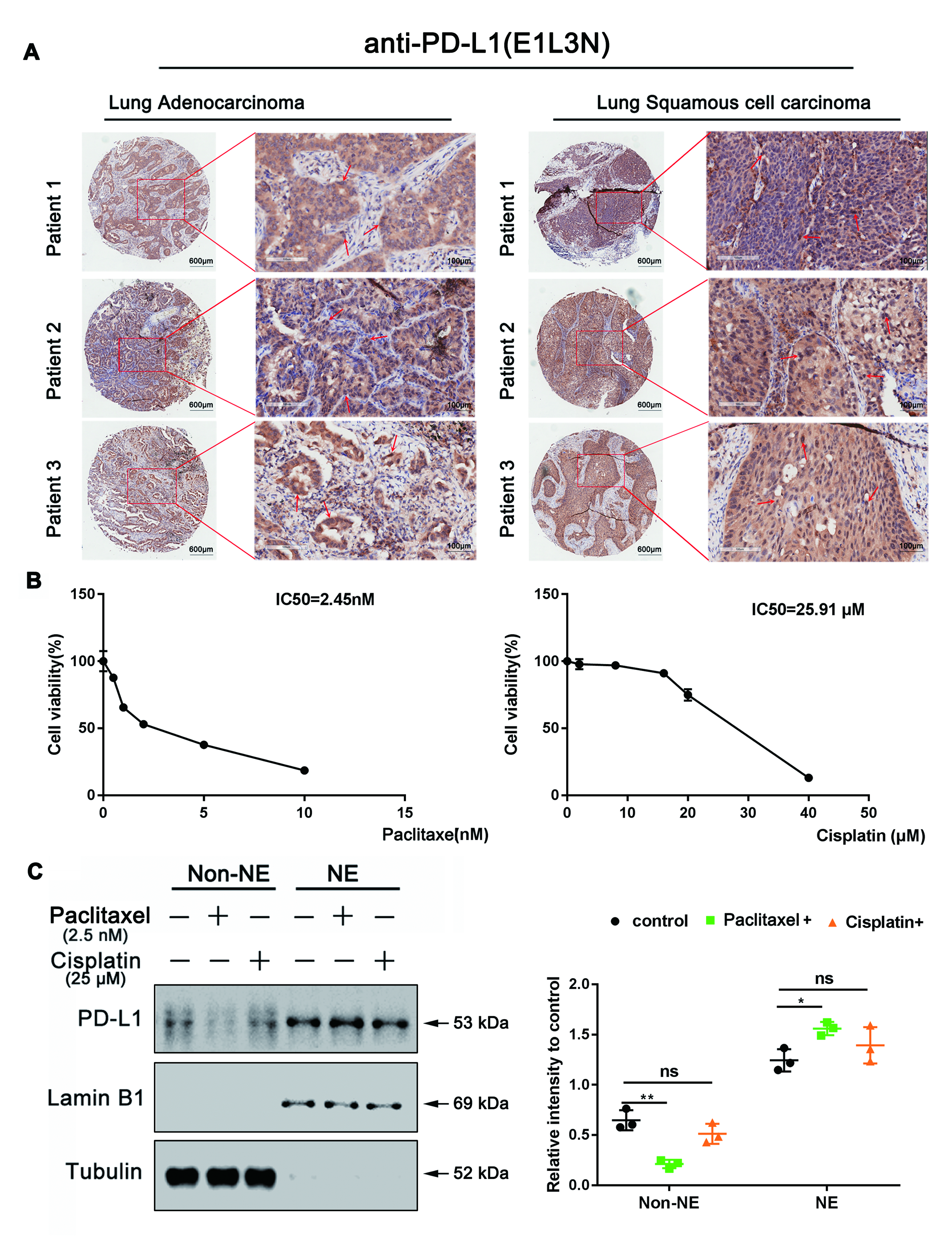

Supplement: Supplementary file 5 — Figure S5 [file 41418_2020_651_MOESM5_ESM.tif]

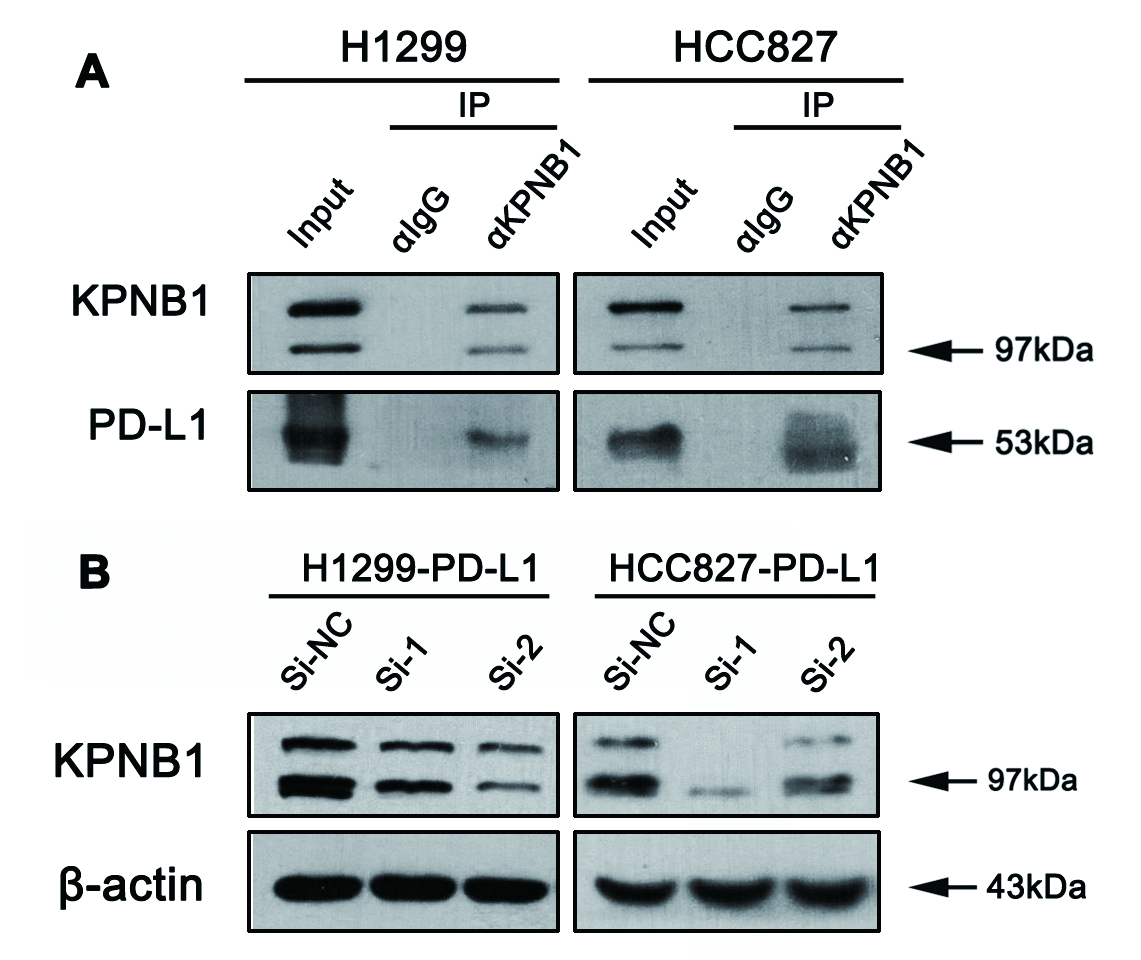

Supplement: Supplementary file 6 — Figure S6 [file 41418_2020_651_MOESM6_ESM.tif]

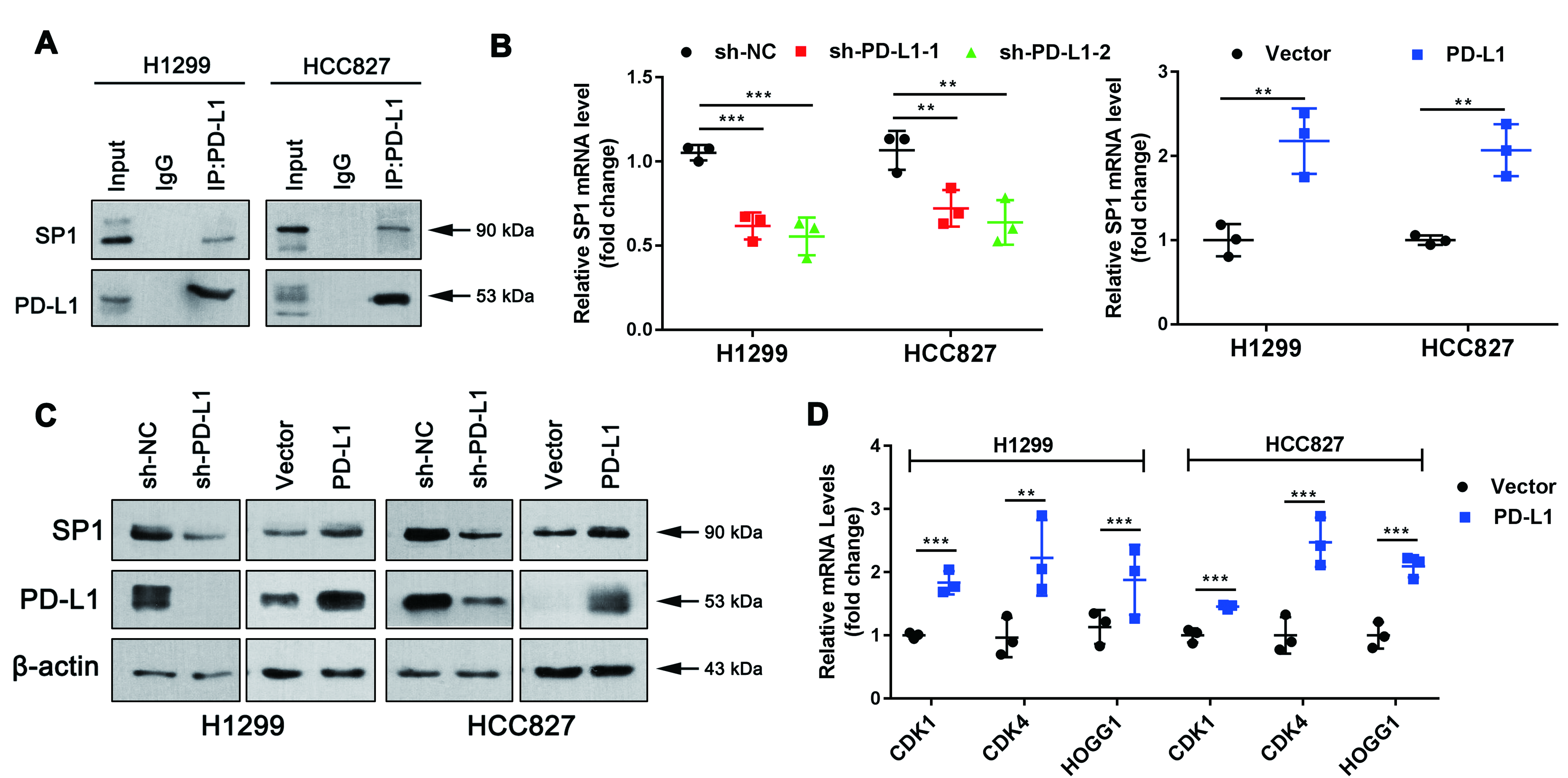

Supplement: Supplementary file 8 — Figure S8 [file 41418_2020_651_MOESM8_ESM.tif]
