# Supplementary material for: KPNB1-mediated nuclear translocation of PD-L1 promotes non-small cell lung cancer cell proliferation via the Gas6/MerTK signaling pathway
Source: Cell Death Differ. 2020 Nov 2;28(4):1284–300. doi: 10.1038/s41418-020-00651-5 (PMC8027631; doi:10.1038/s41418-020-00651-5)
Supplement: Supplementary file 13 — Supplementary figure and table legends [file 41418_2020_651_MOESM13_ESM.docx]

**Supplementary figures and tables**

**Table S1 Clinicopathological features of 24 paired NSCLC patients in our study.**

**Table S2 Clinicopathological features of 52 paired NSCLC tissue microarray in our study.**

**Table S3 The sequences of primers used in our study.**

**Table S4 List of signal densities of human RTK phosphorylation Antibody Array (ARY001B, R&D system)**

**Fig. S1 List of signal proteins of human RTK phosphorylation Antibody Array.** Cell lysates from stable PD-L1 knockdown HCC827 cells and control cells were collected and then applied to human RTK protein array. a P-MerTK expression was down-regulated in PD-L1 knockdown cell lines. b A heatmap of all test kinases in HCC827-sh-NC and HCC827-sh-PD-L1 cell lines. c All signal proteins were listed in line with the RTK array. Data were presented as the mean ± SD. Data were analysed using non-paired Student’s t-test. **P < 0.01 vs. control group.

**Fig. S2 Positive and negative controls for PD-L1, p-MerTK and Ki67 staining of IHC assay.** a Positive controls for all these tested proteins. Tonsil was selected for Ki67 staining control; Placenta was selected for PD-L1 positive control. And adrenal gland was selected for p-MerTK positive control. b Negative controls for all tested proteins. All corresponding normal IgG was selected as negative control (Scale bar: 100μm; 50μm).

**Fig. S3 PD-L1 regulates cell cycle and apoptosis to mediate NSCLC cell proliferation via the MerTK signaling pathway.** a PD-L1-overexpressed cells were treated with 3 μM UNC2025 for 48 h and then stained with propidium iodide, followed by flow analysis. The percentages of cells in G0/G1, S and G2/M phases are shown in the histogram. b PD-L1-overexpressed cells were treated with 3 μM UNC2025 for 48 h, and the apoptotic cells were labelled by Annexin V and PI. Data were presented as the mean ± SD. Data were analysed using two-way ANOVA analysis followed by Bonferroni's post hoc test. ***P < 0.001 vs. control or as indicated.

**Fig. S4 PD-L1 promotes Gas6 secretion independent of autophagy and the protein transport pathway.** PD-L1-overexpressed H1299 and HCC827 cells were starved with medium containing 1% FBS for 12 h and then treated with 50 nM DMA, 1 mM 3-MA or 10 ng/ml BFA for 24 h. The supernatant was collected for ELISA. Data were presented as the mean ± SD. Data were analysed using non-paired Student’s t-test. **P < 0.01 vs. control group.

**Fig. S5 Paclitaxel treatment can induce PD-L1 nuclear translocation.** a Tissue microarray analysis of nPD-L1 expression with anti-PD-L1 antibody staining (E1L3N, Cell Signaling Technology) in 52 paired NSCLC tissues. Among them, 3 lung adenocarcinoma cases and 3 squamous cell carcinoma cases were selected as representative images. And the red arrows indicated positive nPD-L1 staining. b The IC50 values of paclitaxel and cisplatin in HCC827-PD-L1 cell lines. c Cellular fraction was obtained and western blot assay of cytoplasmic and nPD-L1 expression after paclitaxel and cisplatin treatment. The Relative quantification of PD-L1 expression was shown in the right dot plots. Data were presented as the mean ± SD. Data were analysed using one-way ANOVA analysis followed by Bonferroni's post hoc test. *P < 0.05; **P < 0.01vs. control group or as indicated. ns, not statistically significant.

**Fig. S6 KPNB1 interacted with PD-L1 in NSCLC cell lines.** a The endogenous interaction between PD-L1 and KPNB1 via Co-IP assay. b Western blot assay of KPNB1 expression after KPNB1 knockdown with two separate siRNAs in PD-L1-overexpression cells.

**Fig. S7 Increased KPNB1 expression in NSCLC tissues is positively related to poor survival and advanced TNM stage.** a KPNB1 was over-expressed in lung cancer tissues. b, c Kaplan-Meier survival curves indicated that higher KPNB1 expression in NSCLC tissues was associated with poorer OS and PFS probability. d Over-expressed KPNB1 indicated poor overall survival based on the LinkedOmics database. e Advanced TNM stage was correlated with higher KPNB1 expression.

**Fig. S8 PD-L1 regulates Sp1 expression.** a Co-IP assay of PD-L1 and Sp1 interaction in H1299 and HCC827 cells. b,c The mRNA and protein levels of Sp1 after PD-L1 knockdown or overexpression. d The mRNA levels of *CDK1*, *CDK4* and *HOGG1* in PD-L1-overexpressed cells. Data were presented as the mean ± SD. Data were analysed using non-paired Student’s t-test, one-way or two-way ANOVA analysis followed by Bonferroni's post hoc test. **P < 0.01, ***P < 0.001 vs. control or as indicated.
