# Supplementary material for: KPNB1-mediated nuclear translocation of PD-L1 promotes non-small cell lung cancer cell proliferation via the Gas6/MerTK signaling pathway
Source: Cell Death Differ. 2020 Nov 2;28(4):1284–300. doi: 10.1038/s41418-020-00651-5 (PMC8027631; doi:10.1038/s41418-020-00651-5)
Supplement: Supplementary file 14 — Suplementary materials and methods [file 41418_2020_651_MOESM14_ESM.docx]

**Immunohistochemical (IHC) staining**

NSCLC tissue and the corresponding adjacent tissues were fixed in 10% formalin and then embedded in paraffin. Consecutive paraffin sections (4 μm) were deparaffinised in xylene, rehydrated through graded ethanol solutions, and washed in distilled water. Antigen was retrieved by heating sections for 20 min at 95℃ using EDTA solution (PH 11.0) for p-MerTK, or citrate buffer (PH 6.0) for PD-L1 and Ki67, followed by cooling for 20 min at room temperature. Endogenous peroxidase activity was quenched by incubation in 3% H_2_O_2_ in H_2_O for 5 min. After blocking with 2% normal serum, sections were washed and incubated with primary antibodies at 4℃ overnight, including anti-PD-L1 (1:1000 dilution, 2B11D11, ProteinTech Group Inc., Chicago, IL, USA), anti-p-MerTK (1:50 dilution, ab192649, Abcam, Cambridge, UK) and Ki67 antibody (1:200 dilution, 8D5, Cell Signaling Technology, Danvers, MA). In some conditions, sections were incubated with anti-PD-L1 (1:50 dilution, 28-8, Abcam, Cambridge, UK), or anti-PD-L1 (1:200 dilution, E1L3N, Cell Signaling Technology, Danvers, MA) separately. After washing, slides were incubated for 30 min with biotinylated anti-rabbit antibody for p-MerTk and Ki67, or anti-mouse antibody for PD-L1 (1:200 dilution; Vector Laboratories). Signal was developed using DAB working solution kit (ZL1-9019, ZSGB-BIO, China) per the manufacturer’s protocol. Finally, the tumor sections were stained with hematoxylin and evaluated under an Olympus IX73 microscope (Olympus USA, Center Valley, PA, USA).

In addition, we also set negative and positive controls in our experiment. For PD-L1 staining, tonsil was selected for positive control. P-MerTK was highly expressed in endocrine glands like adrenal gland, parathyroid gland. So we set adrenal gland as positive control for p-MerTK. And tonsil was chosen for ki67 positive control. All negative controls were performed using isotyppe IgG. In addition, we also choose tissues that nearly contained no target protein expression level as negative control. For PD-L1, we selected adrenal gland. For Ki67, we selected adrenal gland, and skeletal muscle as set as negative control for p-MerTK.

**Plasmid construction and bacterial transformation**

The cDNA encoding PD-L1 and Sp1 were amplified and cloned into pCDH-CMV-Flag -Puro vector using the endonucleases EcoRI and XbaI. For KPNB1-overexpressed plasmids, KPNB1 cDNA was amplified and cloned into pcDNA3.1-Myc vector using Swal and Not1 endonucleases. Then the recombinant plasmids were run on gels and the correct ones were selected for further bacterial transformation. The 100ng constructed DNA was mixed into 50μl competent cells and incubated for 30 min on ice, followed by 42°C water bath for 90 secs and 2 min on ice. After added with 250μl LB media without antibiotic and grow in 37°C shaking incubator for 45 min, some mixture of the transformation was plated onto the LB agar plate with antibiotic for positive clones’ selection. Then the selected clones were amplified and extracted from LB liquid medium using the Mini plasmid extraction kit (D6942-02, Omega Bio-TEK). All extracted clones were sequenced and correct ones were used for further construction of stable cell lines.

**The establishment of stable PD-L1 or KPNB1-overexpressing cell lines**

For the construction of stable PD-L1-overexpressing cell lines, the coding sequence of PD-L1 was subcloned into a pLVX-IRES-Neo vector using the endonucleases EcoRI and XbaI for expression by a Lenti-X lentiviral expression system (Clontech, Mountain View, CA, USA).

For the construction of KPNB1-overexpressing cell lines, a 2619-bp fragment of the KPNB1 coding sequence was synthesized (Genewiz, Suzhou, China) and subcloned into the PLVX-IRES-Neo vector using the endonucleases SwaI and NotI. The empty vector was used as a negative control. The PD-L1or KPNB1-overexpressing construct were individually co-transfected with the packaging plasmids (MDL: VSVG: REV=5:3:2) into HEK 293T cells using Lipofectamine 2000 (Invitrogen, Waltham, MA, USA). HEK 293T cells were cultured in DMEM medium for additional 48h. The supernatant was collected and filtered with 0.45μm membrane. Then the HEK 293T cells were added with 10 ml fresh medium to culture for another 24h to collect more packaged lentiviruses. When H1299 and HCC827 cells were at the logarithmic growth state, 2ml lentiviruses were used to infect cells. 2 days later, the cultured supernatant were replaced with fresh medium and the infected cells were selected with 400μg/ml G418 (Amresco, Solon, OH, USA).The coding sequences were summarized in Supplementary table 4.

**The establishment of stable PD-L1-knockdown cell lines**

The DNA fragment (PD-L1 ShRNA-1, 5′-GCATTTGCTGAACGCATTTAC-3′; PD-L1 ShRNA-2, 5′-CGAATTACTGTGAAAGTCAAT-3′) was subcloned into the pGV2-U6-Puro (GenePharma, Shanghai, China) containing the endonucleases BamHI and EcoRI. Similar to methods above, the PD-L1-silenced construct or the negative control was co-transfected with packaging plasmids into HEK 293 T cells using Lipofectamine 2000 (Invitrogen). After 48 h, the cells were infected with the packaged lentiviruses and cultured for 2 days before being selection with 2 μg/ml puromycin (Sigma-Aldrich, St. Louis, MO, USA)

**The establishment of stable KPNB1-knockout cell lines**

To establish stable KPNB1-knockout cell lines, guide RNA (gRNA) sequences were synthesized (Genewiz). The target sequences of the KPNB1-gRNA were as follows: Forward: 5'-CACCGACCGTGTCTCCCGATCGGC-3' and Reverse: 5'- AAACGCCGATCGGGAGACACGGTC-3'. Then, we subcloned the KPNB1-gRNA into the lentiviral vector Lenti-CRISPR v2 (GenePharma, Shanghai, China) digested with BsmBI after phosphorylation and annealing. The right Lenti-CRISPR-sgKPNB1 plasmid was confirmed by sequencing. Then the above plasmid or empty vector was co-transfected with packaging plasmids into HEK293T cells using Lipofectamine 2000 (Invitrogen). After incubation, the packaged lentiviruses were collected and used to infect H1299 and HCC827 cells. After 48 h, stable cells were selected with 2 μg/ml puromycin (Sigma-Aldrich, St. Louis, MO, USA).
